# Hi MagicRing, tell me where I am: Toward affordable, physically reliable 3D plant phenotyping with MobilePheno3D

**DOI:** 10.1016/j.abiote.2026.100045

**Published:** 2026-03-28

**Authors:** Yuhui Zheng, Lu Gao, Jiafei Zhang, Liming Miao, Hongfang Zhu, Xuedong Yang, Dingyu Zhang, Zhiguo Han, Xiaofeng Li, Weimin Zhu

**Affiliations:** a Shanghai Key Laboratory of Protected Horticulture Technology, Horticultural Research Institute, Shanghai Academy of Agricultural Sciences, Shanghai, 201403, China; b MetaPheno Laboratory, Shanghai, 201114, China; c PhenoTrait Technology Co., Ltd., Beijing, 100096, China

**Keywords:** 3D plant phenotyping, Metric-scale recovery, 3D reconstruction, Affordable phenotyping, MagicRing

## Abstract

3D plant phenotyping has garnered significant interest for its ability to quantify key structural traits such as plant volume and canopy architecture. However, standard monocular 3D reconstruction techniques suffer from inherent scale ambiguity, requiring an additional step to recover the true metric scale of the plants. Existing scale recovery methods, whether based on precisely fabricated 3D objects or planar patterns such as checkerboards, have been successfully applied in controlled environments but face practical constraints in certain real-world scenarios: some require costly fabrication or pre-reconstruction calibration, which can limit throughput in dynamic field environments. Here, we present MagicRing, a novel, affordable, and physically reliable post-reconstruction scale recovery approach that addresses these specific constraints and provides a complementary solution for high-throughput, mobile, and field-based phenotyping. MagicRing features a simple red ring printed on A4 paper with a known diameter. By leveraging color-based segmentation and geometric curve fitting, our approach automatically detects the ring within 3D point clouds, recovers the metric scale, and establishes a standardized world coordinate system without the need for pre-calibration. Its planar, isotropic design ensures robustness even under significant occlusion. We demonstrate the utility of MagicRing through MobilePheno3D, an integrated smartphone-based pipeline that performs fully automated 3D reconstruction, scale recovery, and phenotypic extraction from video sequences. This system, which was validated across multiple plant species, including vegetables, wheat, rice, and maize in both indoor and field settings, reliably reconstructs aboveground and root structures and supports continuous growth monitoring. MagicRing decouples data collection from data analysis, enabling a workflow transition from conventional step-by-step, scene-specific calibration toward more scalable, high-throughput 3D plant phenotyping.

*Dear Editor*,

Plant phenotyping underpins modern breeding, precision crop cultivation, early stress detection, and intelligent farm management by quantifying information about morphology, physiology, and biochemistry across the plant lifecycle [1]. Three-dimensional (3D) plant phenotyping has received particular interest in recent years because it enables direct measurement of plant volume, canopy structure, and other biomass information lost in 2D imaging [2,3]. However, standard monocular 3D reconstruction techniques, including Structure from Motion (SfM) [4] and Multi-View Stereo (MVS) [5], exhibit inherit scale ambiguity such that reconstructed 3D plants only reflect the relative scale. To acquire realistic 3D phenotypes, it is essential to recover the metric scale of the plant.

At the core of scale recovery is a procedure called camera calibration, which estimates the camera pose for each view based on intrinsic parameters (focal length, principal point, distortion) and extrinsic parameters (rotation and transition matrices). Extrinsic parameters can help transform the camara coordinate system into the world coordinate system, thus recovering the metric scale. There are two major approaches for scale recovery: 3D object calibration and planar pattern calibration.

3D object calibration typically employs a precisely manufactured object with known metric coordinates, such as a cube or an array of non-coplanar points. This approach directly generates a representation of the projective geometry of the camera model; a single image containing the full object can, in principle, provide sufficient constraints to solve for all parameters. By contrast, the dominant paradigm, planar pattern calibration, pioneered by Zhang [6], utilizes a 2D planar pattern such as a checkerboard. By capturing this planar from a diversity of viewpoints, a series of homographies (plane-to-image projections) are obtained. Through nonlinear optimization, these constraints are jointly solved to yield the camera pose for each view.

The suitability of each paradigm is contingent upon the specific constraints and objectives of the phenotyping environment. 3D object calibration offers logistical simplicity, as it can be used for static, multi-camera setups common in greenhouse or growth chamber imaging cabinets [[7], [8], [9], [10]]. A single, synchronized capture of a dedicated 3D fixture can establish a global coordinate system for all sensors simultaneously, streamlining the initial calibration step. However, this approach has significant limitations. The fabrication of metrologically accurate 3D objects is costly and cumbersome. More importantly, due to its physical intrusion, 3D object calibration is poorly suited for dynamic, in-field phenotyping platforms or for the frequent recalibration needed due to sensor drift or reconfiguration. The occlusive nature of plant canopies also often obstructs the full view of a 3D object placed within a scene.

Consequently, the planar pattern paradigm has become the *de facto* standard for plant phenotyping. Its primary advantage is flexibility: a lightweight, portable planar calibration board can be easily introduced into complex scenes, such as within a plant canopy, and imaged from various angles without obstructing the plants. This facilitates robust on-site, periodic recalibration. This method requires more images and computational bundling than 3D object calibration. However, the use of a robust planar calibration board, such as a checkerboard [11] or ArUco boards [12], which combine a chessboard with fiducial markers, ensures reliable feature identification even under partial occlusion by leaves or stems.

However, for certain application scenarios, particularly mobile, field-based phenotyping, planar-based approaches may face practical challenges in scaling the throughput. First, unlike post-reconstruction approaches that recover metric scale and coordinate orientation directly from the reconstructed point cloud, planar-based pre-reconstruction approaches require detecting calibration objects in captured images and embedding the results into the multi-view reconstruction process. This inseparable coupling between calibration and reconstruction hinders large-scale batch processing. Second, some methods can fail when significant occlusion occurs due to the reliance on accurate corner detection [13,14], which increases uncertainty in dynamic, field-based environments. Third, most planar-based approaches recover only the metric scale but still require additional human intervention or sophisticated postprocessing to build the world coordinate system for phenotype extraction. Fourth, deploying a multi-camera system remains costly and unrealistic in the wild. Hence, 3D plant phenotyping awaits a robust, affordable scale recovery approach that is suitable for large-scale 3D reconstruction, resilient to heavy occlusion, and easy to implement under different imaging conditions.

Here, we introduce MagicRing, a novel post-reconstruction approach for metric-scale recovery. As shown in Fig. 1A, MagicRing is a specially designed red ring printed on A4 paper with a preset diameter (Fig. S1). Placing the MagicRing with a plant to be reconstructed can inform the reconstruction plane and function as a known-size 3D object to assess the physical size of the plant. The key idea is to jointly exploit color-based segmentation and geometric curve fitting to localize and recover the red ring from 3D point clouds. Since the exact diameter of the ring is known, it is easy to recover its metric scale and therefore the plant scale as well (Fig. 1C). The planar nature of MagicRing also facilitates the automated generation of the coordinate system, which simplifies subsequent phenotypic analysis (Fig. 1D). Thanks to the isotropic properties of MagicRing, it can function even under rather heavy occlusion (Fig. 1B; b4). Table 1 compares the advantages and disadvantages of MagicRing against other off-the-shelf scale recovery approaches. Despite its simplicity, MagicRing combines the advantages of both planar pattern calibration and 3D object calibration.

**Fig. 1:**
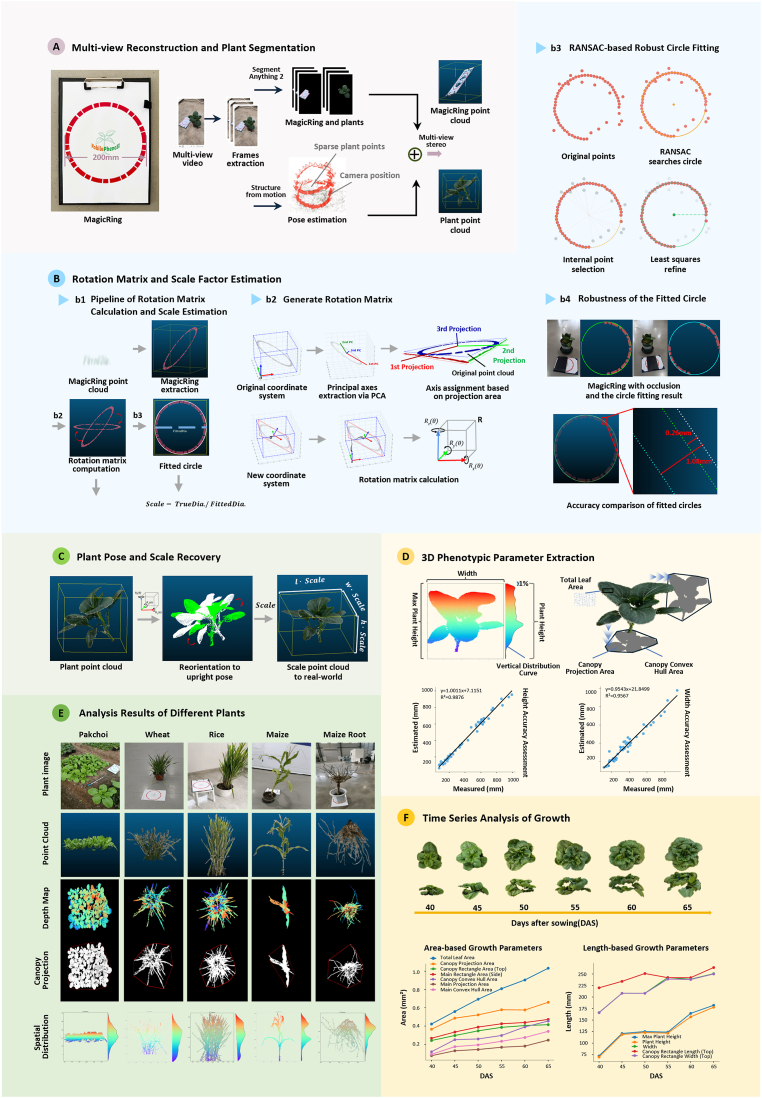
From visual reconstruction to quantitative analysis: A MagicRing-guided pipeline for point cloud calibration and phenotypic parsing of plants. This figure illustrates the automated processing pipeline utilizing MagicRing for high-precision dynamic phenotype extraction from raw video data. **A** The workflow begins with frame extraction and pose estimation from multi-view videos; the generated raw plant and MagicRing point clouds provide the morphological foundation for subsequent analysis. **B** To recover the true orientation and scale of the plant, a new coordinate system is established via Principal Component Analysis (PCA) and axial projection (b2). Simultaneously, a robust RANSAC-based algorithm is employed for precise circle fitting to the MagicRing point cloud, ensuring reliability even under noisy or occluded conditions (b3, b4). Leveraging the geometric priors provided by MagicRing, the rotation matrix and scale factor are accurately computed (b1). **C** This enables the spatial reorientation and isometric scaling of the original plant point cloud, restoring it to its true-to-life orientation and physical dimensions. **D** Once the metric-scale point cloud has been obtained, key morphological parameters are quantitatively assessed, including leaf area, projection area, and plant height distribution. **E, F** This approach demonstrates good generalizability across crop species and for root system reconstruction (E), enables the precise recording of morphological evolution through long-term monitoring and reveals the dynamic patterns of phenotypic parameters (F).

**Table 1:** Comparison of different scale recovery approaches. Different symbols denote the performance level or feature capability of each method. -: Poor/Not Supported; +: Fair/Partial Support; ++: Good; +++: Excellent/Strong Advantage.

Comparison dimension	Checkerboard	ArUco	ChArUco	AprilTag	Sphere	Cube	Pot	MagicRing
Processing Stage	Pre-Recon	Pre-Recon	Pre-Recon	Pre-Recon	Post-Recon	Post-Recon	Post-Recon	Post-Recon
Occlusion Robustness	-	+	++	++	-	+	+	+++
Lighting Robustness	++	-	+	++	+	+	+	++
Corner Dependency	++	-	++	++	-	-	-	-
Requires Encoding	-	++	++	++	-	-	-	-
Fabrication Difficulty	++	++	++	-	-	-	-	++
Auto Coordinate System	-	++	++	++	-	-	-	+++
Automation Level	+	+	+	+	-	-	-	+++
Fitting Complexity	-	-	-	-	-	-	++	++
Ease of Segmentation	++	+	+	++	-	-	-	++
Cost Efficiency	++	++	++	+	-	-	++	++

To test the utility of MagicRing, we employed MobilePheno3D, a smartphone-based 3D plant phenotyping tool. By simply capturing a video sequence around a plant, MobilePheno3D enables fully automated 3D reconstruction, metric-scale recovery, and 3D phenotypic analysis. Through several case studies (Fig. 1E), we demonstrated the ability of MobilePheno3D for cross-species (pak choi, wheat, rice, and maize), cross-scene (indoor and field), and cross-organ (leaves and roots) reconstruction. This tool can also reliably trace the growth status of the plant (Fig. 1F). MobilePheno3D represents the first unified approach for capturing video sequences and using them for 3D phenotype extraction. Since MagicRing decouples data collection from data analysis, MobilePheno3D represents a methodological advancement from conventional step-by-step, scene-specific 3D reconstruction toward more automated, high-throughput 3D plant phenotyping for smartphone-based field applications.

## 1 Materials and methods

### 1.1 Plant materials and video acquisition

Consumer-grade smartphones were employed in conjunction with MagicRing markers (Fig. S2) to capture video sequences on a diverse range of crop species and organs, including pak choi, wheat, rice, and maize plants and maize roots (Fig. S3). Manual measurements of key morphological traits (e.g., plant height and canopy width) were performed to compare the automated measurements. Potted pak choi was chosen for time-series phenotypic analysis. Videos were recorded at 40, 45, 50, 55, 60, and 65 days after sowing (DAS), spanning from the vegetative to reproductive growth stages.

### 1.2 MagicRing-empowered MobilePheno3D

Our approach includes four stages: multi-view reconstruction and plant segmentation, rotation matrix and scale estimation, metric-scale point cloud recovery, and phenotypic parameter extraction.

### 1.3 Multi-view reconstruction and plant segmentation

Our pipeline begins with the acquisition of a 360° wrap-around video of the plant with the MagicRing reference marker. SfM is performed to estimate camera poses and generate sparse point clouds of the plant. Segment Anything Model 2 (SAM2) [15] is then employed to segment plant instances from MagicRing, followed by the standard MVS algorithm used to reconstruct dense point clouds for the plant and the MagicRing, which reside within a unified relative coordinate system.

### 1.4 Rotation matrix and scale factor estimation

The geometric characteristics of MagicRing are leveraged to eliminate the scale ambiguity in MVS reconstruction. This process involves three steps: i) construction of a PCA-based coordinate system, ii) robust circle fitting, and iii) scale factor computation. By applying PCA to the MagicRing point cloud, the three primary eigenvectors represent the three axes of the new coordinate system. A rotation matrix R∈SO(3) that transforms the coordinate system is then computed. Given the *z*-axis of the old coordinate system $z_{old}$, the rotation matrix that rotates $z_{old}$ to the new *z*-axis $z_{target}$ is computed via Rodrigues’ rotation formula such that

(1) $$R=I+\sin\;\theta \cdot {\left[k\right]}_{\times }+\left(1-\cos\;\theta \right)\cdot {\left[k\right]}_{\times }^{2}\text{,}$$

where

(2) $$\begin{array}{c} k=\frac{z_{old}\times z_{target}}{∥z_{old}\times z_{target}∥},\theta =arc\;\cos\left(z_{old}\cdot z_{target}\right)\text{,} \end{array}$$

and ${\left[k\right]}_{\times }$ is the skew-symmetric matrix of the rotation axis *k.* The MagicRing point cloud is then projected onto a 2D plane, followed by two-stage circle fitting: the RANSAC algorithm first searches for an initial circle, and a least-square refinement is then executed on the inliers to obtain a high-precision fitted radius *r*; thus, the reconstructed diameter $D_{fitted}=2r$. Since the true diameter of the MagicRing $D_{true}$ is known (200 mm), the scale factor *s* takes the form ${s=D_{true}/D}_{fitted}$. The scale factor can convert the point units in the reconstruction coordinate system into the physical unit (millimeters).

### 1.5 Plant pose and scale recovery

With R∈SO(3), we reorientate the plant pose and recover the scale of the original plant point cloud $P_{plant}$ by $P_{plant}^{\prime }=R^{T}P_{plant}$ and $P_{plant}^{\prime \prime }=s\cdot P_{plant}^{\prime }$ , respectively. Through these transformations, the plant point cloud is unified into the standard world coordinate system, possessing true metric scale and vertical pose.

### 1.6 Extraction of 3D phenotypic parameters

Given the recovered metric-scale point clouds, it is easy to derive several 3D phenotypic parameters (Table S1), which can be categorized into three groups: (1) spatial dimensions characterized by the bounding box and vertical distribution statistics of the point cloud, including plant height, canopy width, and vertical distribution curve; (2) area and geometric features such as total leaf area, canopy projection area, and main projection area; and (3) morphological envelopes including plant compactness and spatial occupancy characteristics.

### 1.7 Robustness validation of MagicRing

To evaluate the suitability of MagicRing for use in challenging environments, a systematic robustness analysis was conducted focusing on four critical factors: varying illumination conditions, background complexity, ring inclination, and ring occlusion (Fig. S4–S8).

## CRediT authorship contribution statement

**Yuhui Zheng:** Writing – review & editing, Writing – original draft, Methodology. **Lu Gao:** Writing – original draft, Investigation. **Jiafei Zhang:** Writing – review & editing, Writing – original draft, Visualization. **Liming Miao:** Validation, Resources, Investigation. **Hongfang Zhu:** Writing – review & editing, Investigation, Data curation. **Xuedong Yang:** Validation, Investigation, Formal analysis. **Dingyu Zhang:** Writing – review & editing, Validation, Methodology. **Zhiguo Han:** Writing – review & editing, Supervision, Conceptualization. **Xiaofeng Li:** Writing – review & editing, Supervision, Funding acquisition. **Weimin Zhu:** Writing – review & editing, Supervision, Project administration, Conceptualization.

## Declaration of competing interest

The authors declare that they have no known competing financial interests or personal relationships that could have appeared to influence the work reported in this paper.

## Data Availability

Data will be made available on request.
