# Supplementary material for: Hi MagicRing, tell me where I am: Toward affordable, physically reliable 3D plant phenotyping with MobilePheno3D
Source: aBIOTECH. 2026 Mar 28;7(3):100045. doi: 10.1016/j.abiote.2026.100045 (PMC13157090; doi:10.1016/j.abiote.2026.100045)
Supplement: Multimedia component 1 [file mmc1.docx]

**Supplementary Materials**

Hi MagicRing, tell me where I am: Toward affordable, physically-reliable 3D plant phenotyping with MobilePheno3D

Yuhui Zheng^b,c,1^ , Lu Gao^a,1^, Jiafei Zhang^b,c,1^, Liming Miao^a^, Hongfang Zhu^a^, Xuedong Yang^a^, Dingyu Zhang^a^, Zhiguo Han^b,c,^*, Xiaofeng Li ^a,^*, and Weimin Zhu^a,^*

^a^ Shanghai Key Laboratory of Protected Horticulture Technology, Horticultural Research Institute, Shanghai Academy of Agricultural Sciences, Shanghai, 201403, China

^b^ MetaPheno Laboratory, Shanghai, 201114, China

^c^ PhenoTrait Technology Co., Ltd., Beijing, 100096, China

**
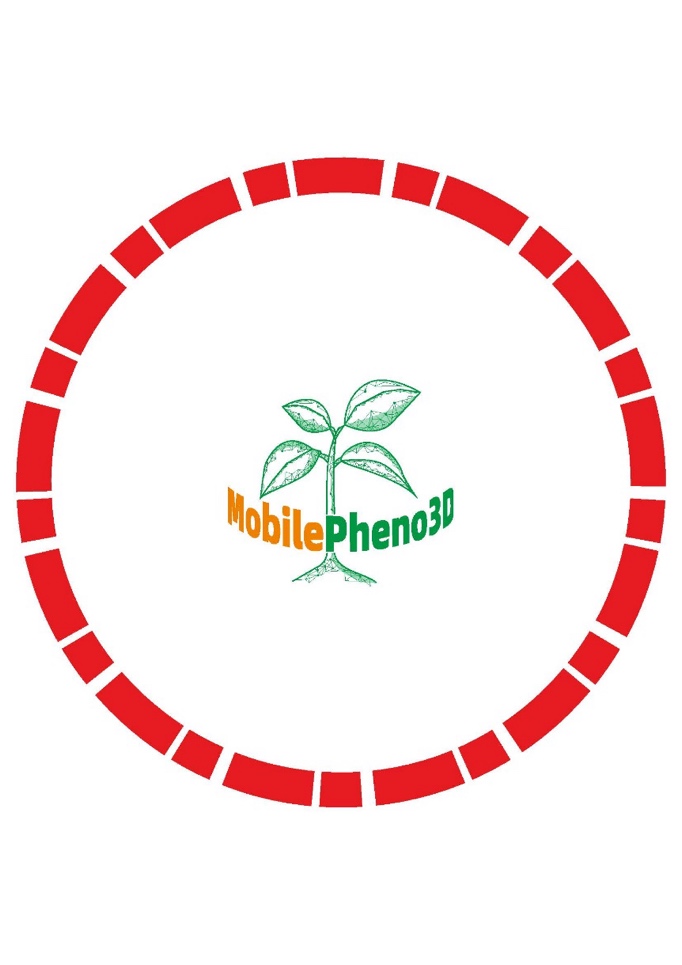
**

**Supplementary Fig. 1 Printable MagicRing Template.** Printable MagicRing template designed for A4 paper.

**
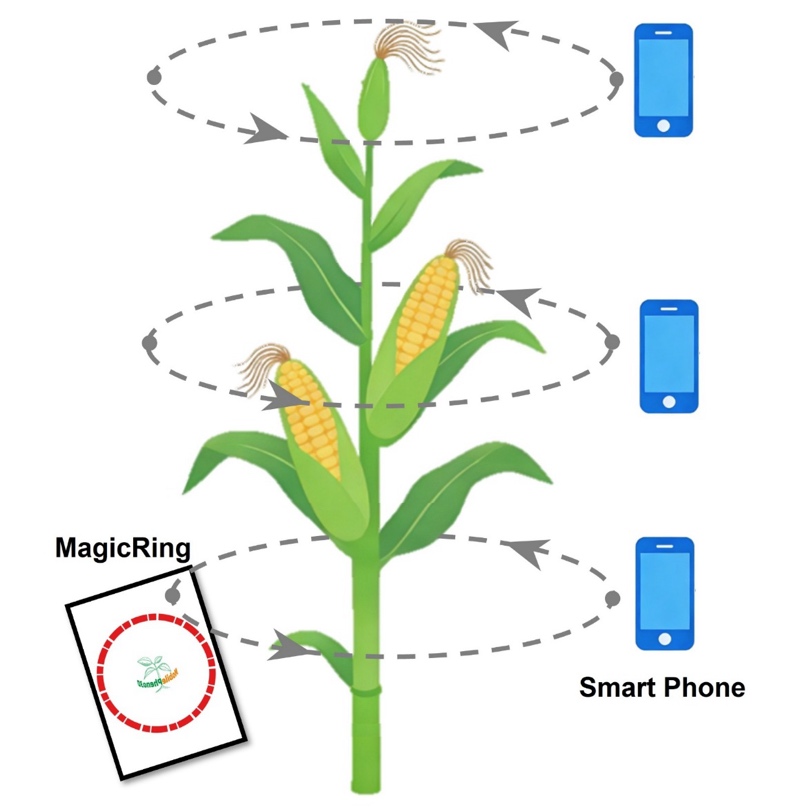
**

**Supplementary Fig. 2 MagicRing Usage.** Detailed method for MagicRing usage and video recording: (1) Print the MagicRing on A4 paper and place it horizontally next to the plant being filmed; (2) Circle the plant once from 2–3 different angles in a single filming session; (3) Avoid shooting too close: capture the whole plant rather than partial views; (4) Keep the video recording within 1–3 minutes with slow and steady filming. This standardized protocol ensures consistent data acquisition across different experimental sessions and users.


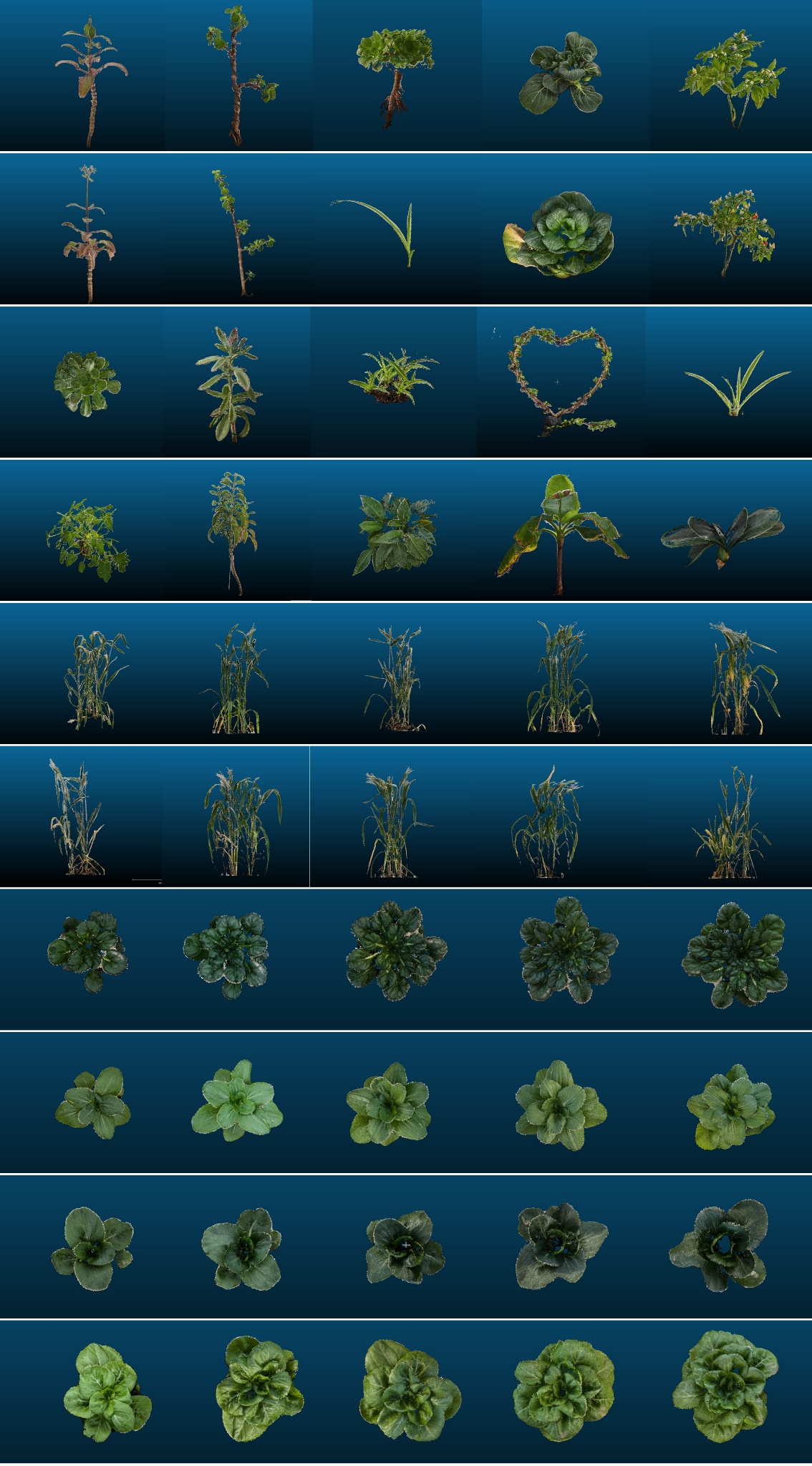


**Supplementary Fig. 3 Reconstruction Results for the Experimental Materials.** Reconstruction results for different plant species under various scene conditions. This figure demonstrates that the reconstructed 3D models preserves fine geometric details and structural integrity for each species tested across diverse environmental settings.


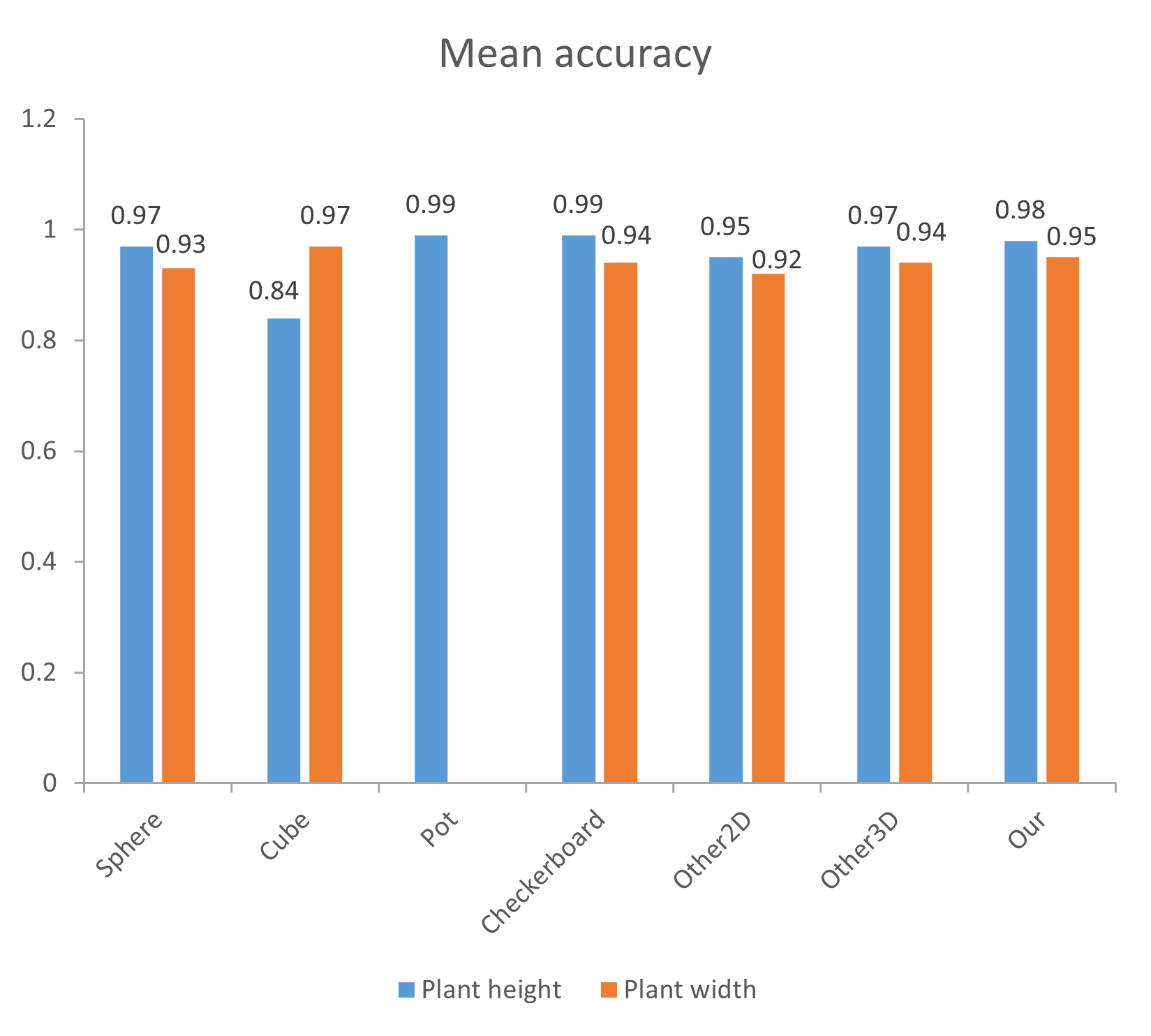


**Supplementary Fig. 4 Comparison of the Accuracy of Scale Recovery Between the MagicRing-based Method and Traditional Methods.** Comparative analysis of scale recovery between the proposed MagicRing-based method and traditional approaches. Quantitative evaluation demonstrated that our method achieved precision comparable to that of established techniques such as checkerboard calibration.


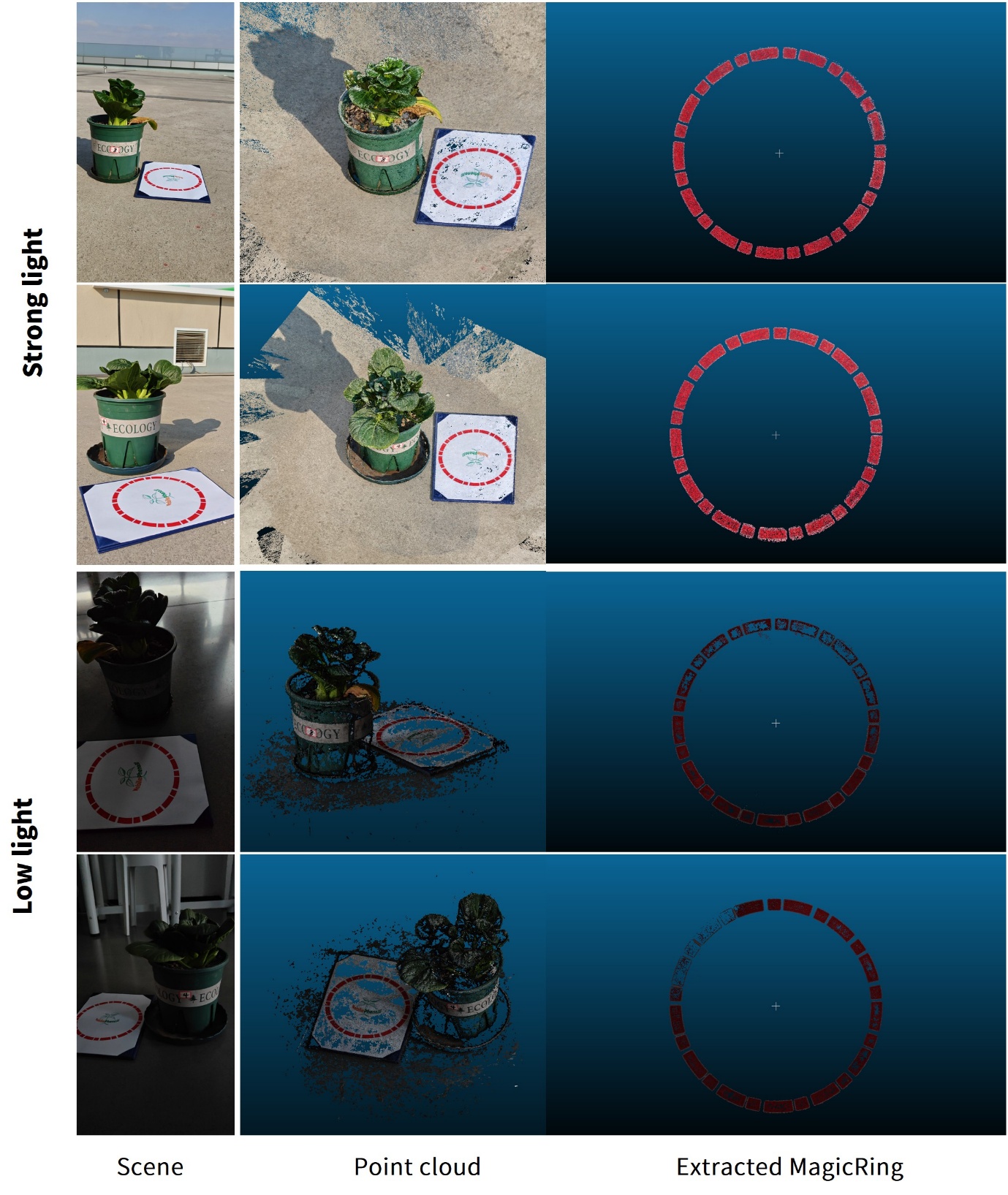


**Supplementary Fig. 5 Robustness Validation: Varying Illumination Conditions.** Reconstruction results under varying lighting conditions, including strong light (intense sunlight) and weak light (dim indoor environments). This validation assessed the method’s reliability across extreme illumination scenarios commonly encountered in field and greenhouse settings. The MagicRing detection and subsequent reconstruction pipeline maintained stable accuracy regardless of changes in ambient lighting, ensuring practical use in uncontrolled real-world environments.


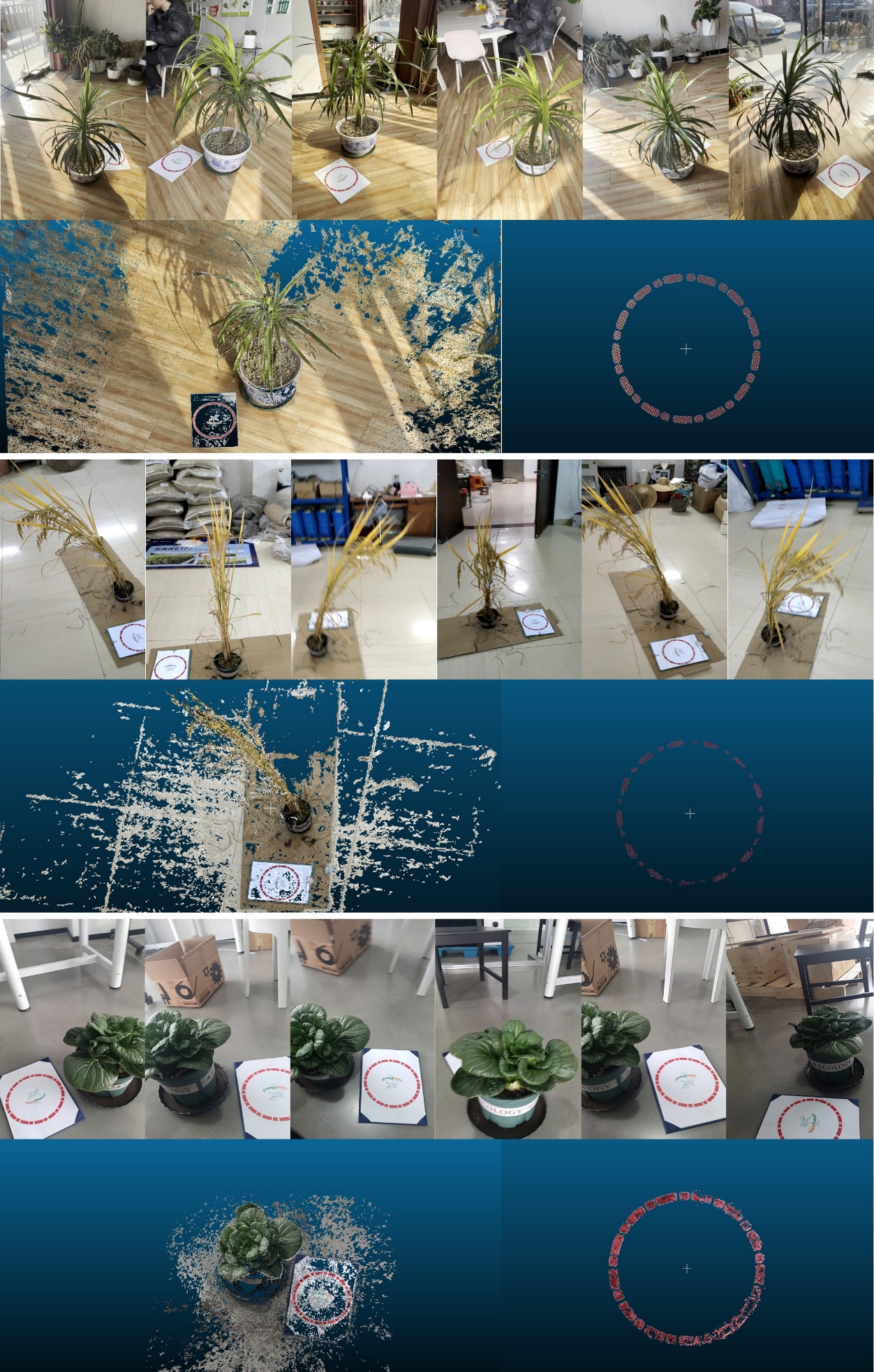


**Supplementary Fig. 6 Robustness Validation: Background Complexity.** Reconstruction results under complex background conditions. This experiment evaluated the method’s discrimination capability when plants are situated against cluttered or visually similar backgrounds that challenge segmentation and feature extraction algorithms. MagicRing’s distinctive circular pattern provides reliable feature points for scale reference even when plant boundaries become ambiguous against complex surroundings.


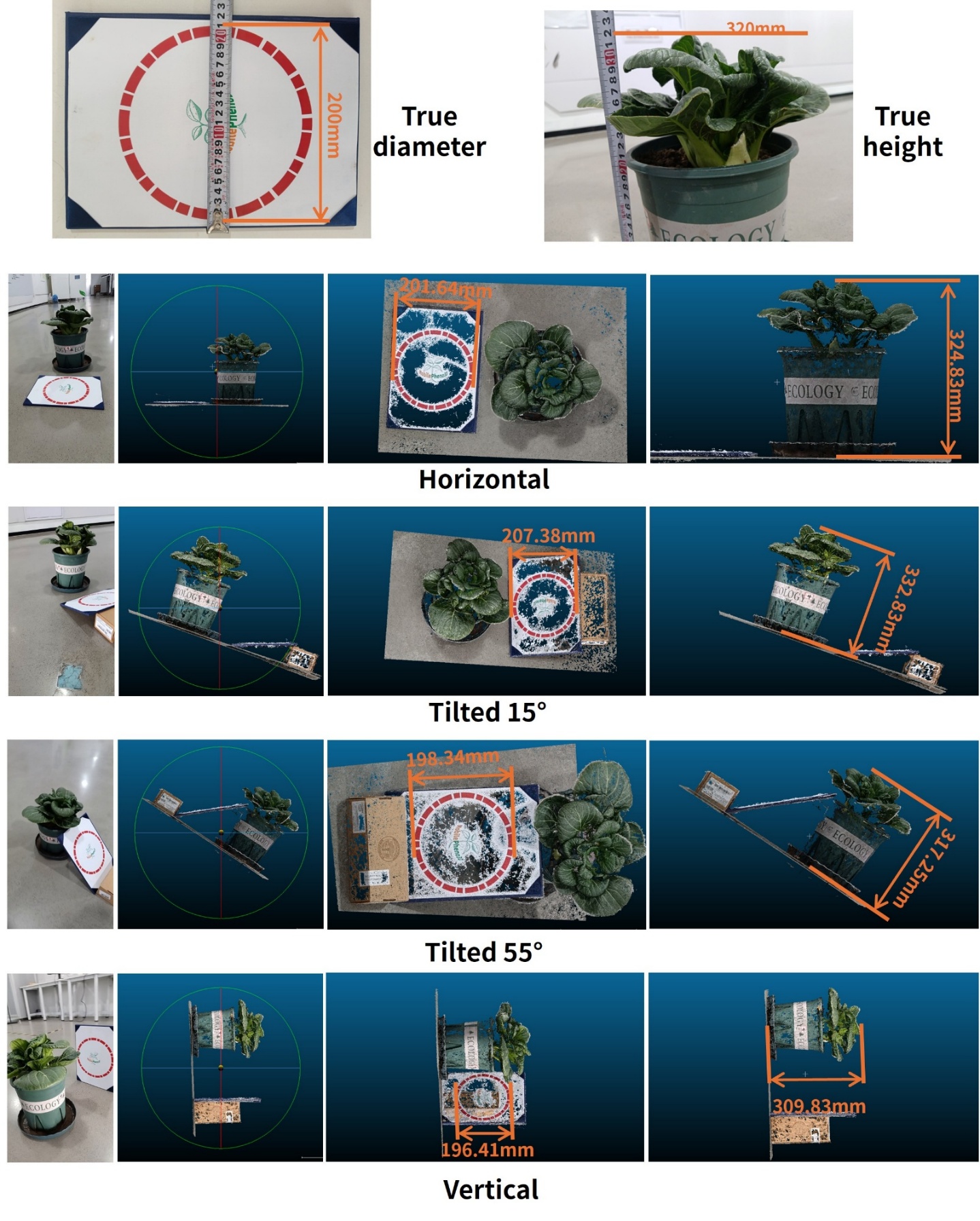


**Supplementary Fig. 7 Robustness Validation: Ring Inclination.** Scale recovery results under varying degrees of inclined MagicRing placement. This analysis examined the geometric stability of the method when the reference ring is not perfectly horizontal due to terrain unevenness, wind disturbance, or placement errors. Systematic tests covered inclination angles ranging from slight tilts (15°) to significant slopes (55°) that might occur during field measurements. The circle-fitting algorithm demonstrated robustness to perspective distortion caused by non-orthogonal viewing angles.


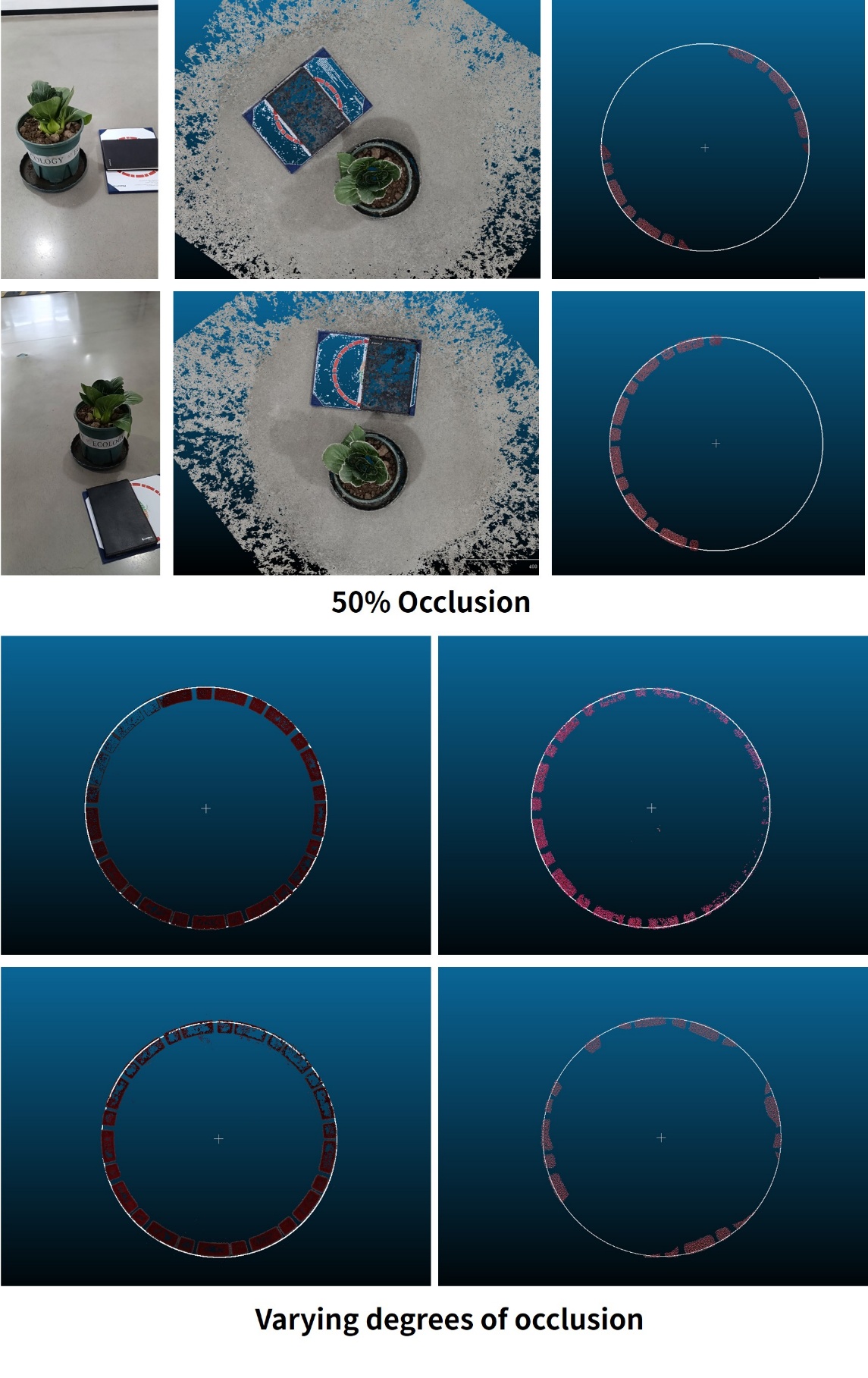


**Supplementary Fig. 8 Robustness Validation: Ring Occlusion.** Circle-fitting results under varying degrees of MagicRing occlusion. This test investigated the method’s tolerance to partial covering of the reference ring by plant leaves, stems, or other environmental objects during filming. Test cases included progressive occlusion levels, from minor coverage to substantial blocking of the ring pattern. Only a slight decrease in accuracy occurred with increasing occlusion, maintaining functional performance even when significant portions of the ring were temporarily hidden.

**Supplementary Table: Detailed description of phenotypic parameters**

| **No.** | **Parameter** | **Description** | **Significance** |
| --- | --- | --- | --- |
| 1 | Max Plant Height | Vertical distance from highest to lowest point | Indicates vertical growth, photosynthesis, yield potential |
| 2 | Plant Height | Avg height of top 1% points minus lowest point | Robust height metric for growth monitoring |
| 3 | Width | Horizontal span (leftmost to rightmost) | Horizontal expansion; affects light capture & density |
| 4 | Total Leaf Area | Sum of triangular facet areas in point cloud | Photosynthetic capacity & biomass accumulation |
| 5 | Bounding Box Volume | Volume of minimal bounding box | Spatial occupancy; aids canopy architecture design |
| 6 | Convex Hull Volume | Volume of the minimal convex polyhedron enclosing the plant point cloud | Represents overall 3D space occupation; indicates canopy compactness; evaluates space utilization efficiency |
| 7 | Canopy Rectangle Length (Top) | Length of min bounding rectangle (top-view) | Canopy horizontal spread; guides planting layout |
| 8 | Canopy Rectangle Width (Top) | Width of min bounding rectangle (top-view) | Canopy coverage; impacts light interception |
| 9 | Canopy Rectangle Area (Top) | Area of min bounding rectangle (top-view) | Canopy coverage & ecosystem productivity |
| 10 | Canopy Projection Area | Projected area from top-view | Canopy coverage & photosynthetic efficiency |
| 11 | Canopy Convex Hull Area | Convex hull area (top-view) | Canopy outer contour; light interception analysis |
| 12 | Main Rectangle Area (Side) | Min bounding rectangle area (main-view) | Lateral shape; wind resistance & stability |
| 13 | Main Projection Area | Projected area from main-view | Lateral space distribution & light use |
| 14 | Main Convex Hull Area | Convex hull area (main-view) | Lateral contour; wind resistance evaluation |
| 15 | Leaf Area Index | Total leaf area per unit ground area | Key index for population photosynthesis & transpiration |
| 16 | Avg Leaf Inclination | Mean leaf tilt angle | Light interception & canopy penetration |
| 17 | Green Ratio | Proportion of green points in point cloud | Plant health indicator |
| 18 | Volume | Voxel count × single voxel volume | Biomass & spatial structure; yield estimation |
| 19 | Height of Max Width | Height layer where width is maximum | Canopy vertical distribution |
| 20 | Vertical Distribution Curve | Fitted curve of point count vs height | Reveals internal canopy layering |
